# Tweaking Photosynthesis: FNR-TROL Interaction as Potential Target for Crop Fortification

**DOI:** 10.3389/fpls.2020.00318

**Published:** 2020-03-24

**Authors:** Hrvoje Fulgosi, Lea Vojta

**Affiliations:** Laboratory for Molecular Plant Biology and Biotechnology, Division of Molecular Biology, Institute Rud¯er Bošković, Zagreb, Croatia

**Keywords:** photosynthesis, linear electron transfer, ROS, electron partitioning, stress-tolerance

Photosynthesis not only supplies plants with needed energy for growth and nutrient storage, but links light-to-chemical energy conversion with redox regulatory networks of the entire cell. By modulating photosynthetic electron flow, plants can adapt to constantly changing light and environmental conditions. Different mechanisms direct electrons formed during light reactions to either energy-conserving or energy-dissipating pathways. Recently described dynamic interactions of the flavoenzyme ferredoxin:NADP^+^ oxidoreductase (FNR) with protein TROL represent elegant molecular switch that can partition electrons downstream of photosystem I. FNR-TROL bifurcation can control energy transfer to either linear flow, which results in NADPH production, or to the rapid electron sink that efficiently prevents reactive oxygen species propagation. Plant genome editing of TROL represents an unexploited alley for improvement of plant stress and defense responses, productivity, and eventually agricultural yield.

The most well-known and established function of plant flavoenzyme ferredoxin: NADP(H) oxidoreductase (FNR) in photosynthetic energy conversion is the synthesis of NADPH (Forti and Bracale, 1984; Carillo and Ceccarelli, 2003; Shin, 2004; Mulo, 2011). This enzymatic reaction utilizes two molecules of reduced ferredoxin (Fd) to produce one molecule of NADPH (Arakaki et al., 1997; Medina and Gómez-Moreno, 2004). This conversion is known as the last step in the linear electron transfer (LET) chain (Allen, 2003; Rochaix, 2011). Generated NADPH is utilized in numerous downstream biochemical reactions, both in chloroplast stroma and in the rest of the cell (Rochaix, 2011; Scheibe and Dietz, 2012). Electron cycling from ferredoxin to NADPH only occurs in the light. In the dark, as well as in non-photosynthetic organisms, the FNR primarily works in reverse, utilizing NADPH to provide reduced ferredoxin for various metabolic pathways, such as oxidative stress response, nitrogen fixation, terpenoid biosynthesis, steroid metabolism, and iron–sulfur protein biogenesis (Aliverti et al., 2008). Thus, FNR links fundamental process of light-to-chemical energy conversion with general plant metabolism (Foyer and Noctor, 2005). The electron donor Fd is a small iron-sulfur protein that acts simultaneously as a hub, and as a bottle neck for the distribution of electrons supplied by photosystem I (PSI) (Paul and Foyer, 2001; Hanke et al., 2004; Balmer et al., 2006; Joliot and Johnson, 2011). FNR binding to thylakoid membranes of vascular plants has been proposed to take place via cytochrome *b*_6_/*f* complex (Clark et al., 1984; Zhang et al., 2001), PSI-E subunit (Andersen et al., 1992), and in a complex with NADPH dehydrogenase (Quiles and Cuello, 1998). However, in all these studies the exact protein association domain responsible for FNR-binding has not been identified. Most recently, the FNR interaction with photosynthetic membranes has also been demonstrated in organisms other than vascular plants (Mosebach et al., 2017; Pini et al., 2019). In a number of essential publications, it has been demonstrated that the distribution of FNR between soluble- and thylakoid-bound states can have profound influence on FAD cofactor assembly (Miyake et al., 1998; Onda and Hase, 2004), Fd-FNR interactions, FNR catalytic properties, oxidative and photo-oxidative stress tolerance, and the regulation of photosynthesis (Palatnik et al., 1997; Rodriguez et al., 2007; Hanke et al., 2008; Peng et al., 2008; Benz et al., 2009, 2010; Jurić et al., 2009; Alte et al., 2010; Yacoby et al., 2011; Twachtmann et al., 2012; Vojta and Fulgosi, 2012; Vojta et al., 2012; Goss and Hanke, 2014; Lintala et al., 2014). Particularly interesting, transgenic plants overexpressing chloroplastic FNR acquire increased tolerance to oxidative stress, while simultaneously displaying normal rates of photosynthesis (Rodriguez et al., 2007). Further, FNR knock-down plants, which have stunted growth and restricted photosynthetic activity, are particularly susceptible to photo-oxidative damage (Hajirezaei et al., 2002; Lintala et al., 2012). Perhaps the most intriguing finding was that the release of FNR from thylakoid membranes could be regulated by oxidative stress, in methyl viologen-dependent fashion (Palatnik et al., 1997). Lastly, in t*ic62trol* double mutants no binding of FNR to thylakoid proteins could be detected, whatsoever (Lintala et al., 2014).

The hypothesis describing additional functions and physiological roles of chloroplastic FNR in vascular plants has been proposed (Benz et al., 2010; Goss and Hanke, 2014). This concepts rest on a series of publications mostly describing binding and release of FNR from the soluble chloroplastic protein Tic62 (Benz et al., 2009; Lintala et al., 2014). Tic62 (62 kDa component of the translocon at the inner envelope of chloroplasts) has initially been discovered as a component of chloroplast inner envelope protein translocation machinery (Küchler et al., 2002), but has subsequently been associated with at least two other compartments, chloroplast stroma, and chloroplast photosynthetic membranes (Balsera et al., 2007). The essential protein region recognized in Tic62 as being responsible for the binding of FNR is present in multiple repeats in many, but not all, Tic62 family members studied (Balsera et al., 2007). The same motif was further identified at the C-terminus of the thylakoid rhodanase-like protein TROL (Jurić et al., 2009). This domain, dubbed membrane recruitment motif (MRM), binds FNR with high efficiency. In Tic62, the interaction was postulated to be light- and pH-dependent (Benz et al., 2009). In 2010, Benz et al. proposed that the majority of thylakoid-localized FNR is bound to the membrane via two interaction partners, Tic62 and TROL (Benz et al., 2009). They further postulate that soluble form of FNR is sufficient to sustain photosynthetic energy conversion, while the thylakoid pool largely performs other functions. Finally, they suggest that light- and redox-states regulate the distribution of FNR. In the view of that, FNR is stored at the membranes in the dark and during morning hours, thus allowing advantageous flow of electrons to various Fd-dependent pathways (Benz et al., 2009; Vojta et al., 2012). Such scenario could assure proper metabolic adjustments depending to environmental cues.

Soluble Tic62, however, does not contain MRM in all plant species (Balsera et al., 2007), and its attachment points at the thylakoids have yet to be determined. TROL, however, is *bona fide* integral membrane protein with dual localization (Jurić et al., 2009; Vojta et al., 2018). It is present in the mature form in thylakoid membranes, and in the precursor form at the inner chloroplast envelope. TROL is located near PSI, mostly in non-appressed thylakoid regions (Jurić et al., 2009). All known vascular plant TROL sequences contain FNR MRM (Jurić et al., 2009). Additionally, a single MRM of TROL binds FNR with several fold higher affinity then the Tic62 MRM (Jurić et al., 2009). In Arabidopsis, TROL associated with FNR can be isolated in the dynamic supramolecular complex of ~190 kDa (Jurić et al., 2009).

The unique hallmark of TROL is the lumen-located rhodanase-like domain (RHO), which is most likely inactive in a sense of sulfur detoxification, as it contains the aspartate residue instead of the conserved cysteine in the putative active site (Jurić et al., 2009). RHO domains are intriguing due to their ancient origin and structural identity with the catalytic domains of cell-division-cycle (CDC25) dual-specificity phosphatases. Inactive RHO domains are implicated in redox-sensing and were shown to interact with quinolinediones (Brisson et al., 2005). Plant sulfur-transferases and rhodanases have recently been reviewed in two comprehensive papers (Moseler et al., 2019; Selles et al., 2019).

Recently, alternative mechanism of FNR binding and release from TROL, involving redox sensing by the RHO domain, has been put forward (Vojta and Fulgosi, 2012, 2019). According to this model, certain (redox) signal(s) of lumenal origin can be sensed by the RHO and further transduced across thylakoid membrane, resulting in differential FNR binding on the stromal side (Vojta and Fulgosi, 2012). The role of the proline-rich region of TROL, dubbed PEPE, which precedes the MRM, has also been postulated (Jurić et al., 2009). Due to a repeating sequence of turn-inducing proline residues, PEPE might serve as molecular swivel, allowing free movement of bound FNR, or even its alternative associations with various supramolecular complexes, or membrane domains. Such dynamic FNR recruitment might be responsible for alternative partitioning of photosynthetic electrons, and/or prioritization of Fd-dependent pathways. Further, it has been demonstrated that chloroplasts of Arabidopsis *trol* mutants proliferate substantially less superoxide anion radicals then the WT (Vojta et al., 2015). This reduction can be recorded in *trol* chloroplast pre-acclimated to dark and growth-light conditions. Even more remarkable, *trol* chloroplast proliferate almost 40 percent less superoxide anion radicals even in the presence of ROS-generating methyl viologen (paraquat herbicide) (Vojta et al., 2015). These finding are in line with the previously published observations and suggest that FNR permanently detached from TROL can either efficiently scavenge superoxide anion, or that electrons are very rapidly partitioned into certain other pathways(s), different from the LET. In fact, results suggest that LET is preferential only when TROL-FNR association is established (Jurić et al., 2009; Vojta et al., 2015). Alternatively, in the absence of TROL, electrons rapidly flow to other sinks downstream of PSI donor site (Vojta et al., 2015). Finally, it has been demonstrated that in the absence of TROL light- and/or pH-dependent dynamic recruitment of FNR to thylakoids is entirely abolished (Vojta and Fulgosi, 2016). Apparently, TROL-FNR interaction might be the most prominent, if not exclusive, dynamic-type interaction of FNR with photosynthetic membranes of vascular plants.

TROL-FNR interaction could be an entrance to crop improvement *via* various modifications to the interaction itself, or to upstream and/or downstream reactions and pathways. For example, TROL itself could be down- or up-regulated, or its FNR-binding or regulatory domains could be modified by genome engineering. FNR could also be a target of engineering, for example by exchange of FNR enzymes from C4 to C3 plants or *vice versa*. C4 FNR interacts with TROL ITEP domain with many fold higher affinity than the C3 FNR (Rac and Fulgosi, 2019). The increase of TROL levels by itself would not necessarily imply an increase in photosynthetic efficiency, since the improvement would depend on the availability of reduced ferredoxin and the ability of the system to provide the necessary reduction equivalents. However, alterations in the production of reduction equivalents could affect the redox homeostasis of chloroplasts and ultimately produce aberrations instead of plant benefit.

To conclude, we further iterate the concept of photosynthetic membrane recruitment of FNR and emphasize the importance of TROL-FNR dynamic interaction. We posit that TROL-FNR interaction is an important and overlooked mechanism for regulation and prioritization of energy-conserving and energy-dissipating pathways in vascular plant photosynthesis. We propose that TROL interaction with FNR is useful target for genome editing of agriculturally important species, potentially providing more stress-tolerant crops.

## Author Contributions

HF and LV wrote the manuscript.

### Conflict of Interest

The authors declare that the research was conducted in the absence of any commercial or financial relationships that could be construed as a potential conflict of interest.
